# Characteristics of men using non-prescription medicines to treat their lower urinary tract symptoms

**DOI:** 10.1007/s00345-025-05560-1

**Published:** 2025-05-08

**Authors:** Martin C. Michel, Kurt Miller, Tim Schneider, Christian Ude, Matthias Oelke

**Affiliations:** 1 Department of Pharmacology, University Medical Center, Johannes Gutenberg University, Mainz, Germany; 2 Department of Urology, Charité, Berlin, Germany; 3 Praxisklinik Urologie Rhein-Ruhr, Mülheim, Germany; 4 Stern Pharmacy, Darmstadt, Germany; 5 Interdisciplinary Pelvic Floor Center, Kantonsspital Frauenfeld and Münsterlingen, Spital Thurgau AG, Frauenfeld, Switzerland; 6 Hannover Medical School, Hannover, Federal Republic of Germany

**Keywords:** Survey, Over-the-counter medications, LUTS/BPH, Patient preferences, Phytotherapy

## Abstract

**Purpose:**

Many men with lower urinary tract symptoms suggestive of benign prostatic hyperplasia (LUTS/BPH) opt for self-management with non-prescription treatments. We wished to better characterize these men and understand their motivation and experience.

**Methods:**

We conducted an anonymous survey among 477 users of non-prescription treatments for LUTS.

**Results:**

Participating men had a mean age (64.3 ± 7.8 years) and IPSS (17.7 ± 7.9 points) comparable to users of prescription medicines in non-interventional studies, indicating that they were not primarily motivated by mild symptom intensity. They had realistic expectations on efficacy and tolerability. While reasons for preference for a non-prescription treatment varied, 40.0% stated a wish to avoid ‘chemical’ and, vice-versa, to use ‘natural’ treatments. The choice of a specific treatment was largely driven by prior experience, but recommendations from a physician or the pharmacy as well as advertisements also played a role. About two thirds of participants reported to be repeat users and purchasing the same product each time. However, less than a quarter of participants appear to use the non-prescription treatment continuously.

**Conclusions:**

Men self-managing their LUTS/BPH are similar to those using prescription drugs based on age and symptom severity. Their main reasons to prefer non-prescription medicines relate to the wish to avoid ‘chemical’ and the preference for ‘natural’ treatments. Most of them are repeat users of the same product but use it only intermittently.

**Supplementary Information:**

The online version contains supplementary material available at 10.1007/s00345-025-05560-1.

## Introduction

Lower urinary tract symptoms suggestive of benign prostatic hyperplasia (LUTS/BPH) are a prevalent condition in older men and can substantially impair quality of life (QoL). Various prescription medicines are available and guideline-recommended for the medical treatment of LUTS/BPH, including members of the drug classes of α_1_-adrenoceptor antagonists (α-blockers), 5α-reductase inhibitors, and phosphodiesterase type 5 inhibitors. While not approved for the indication of LUTS/BPH, muscarinic receptor antagonists and β_3_-adrenoceptor agonists are used if storage symptoms persist upon treatment with the other drug classes [1–3]. These prescription medicines are available for men with LUTS/BPH with little or no co-pay in most developed countries.

Additionally, various non-prescription medicines are also available for the treatment of LUTS/BPH, often as over-the-counter (OTC) medications. They mostly belong to the group of plant extracts, i.e., are phytotherapeutic agents. This includes saw palmetto (*Serenoa repens* a.k.a. *Sabal serrulata*) berries, pumpkin (*Cucurbita pepo*) seeds and others. Based on the heterogeneity of these preparations such as source of plant materials and methods of extraction, clinical findings from one preparation cannot necessarily be extrapolated to others prepared from the same plant [4]. Therefore, the clinical evidence is very limited for most OTC medications used for the treatment of LUTS/BPH. Based on the lack of robust evidence of effectiveness (not based on evidence that they are ineffective), many LUTS/BPH guidelines do not recommend the use of plant extracts, but some recent guidelines such as those by the Germany Society of Urology recommend specific plant extract preparations for which appropriate clinical data are available [3]. In general, these plant extracts are considered to be well tolerated. However, in contrast to prescription medications, they are not reimbursed by insurance companies in many countries, including Germany.

Men opting for self-management with OTC medications have only been poorly characterized [5]. Specifically, it remains unclear why some men opt to use medications with limited evidence for the treatment of their LUTS/BPH for which they must pay fully out of pocket in contrast to evidence-based prescription medicines that are largely or fully reimbursed. Possible reasons include shame to talk about LUTS with a doctor, considering LUTS to be part of normal ageing, distrust of ‘chemical’ or preference for ‘natural’ treatments, and poor experience with prescription medicines. Therefore, we have performed an anonymous survey among men purchasing OTC preparations for the treatment of LUTS to characterize such men and explore the reasons why they have chosen certain preparations.

## Patients and methods

### Overall study outline

We performed an anonymous, non-interventional survey among men who had purchased a non-prescription medication in a public pharmacy for the treatment of their own LUTS/BPH. The study protocol was finalized on 20th August 2020, i.e., before the first patient-completed questionnaire was received. The study was divided into a pilot and a main phase of similar design. The pilot study was designed to test the feasibility and usefulness of the questionnaire. If needed, a revision of the questionnaire was to be conducted. The main phase was designed to use the final version of the questionnaire. If no major revision of the questionnaire was needed after the pilot phase, data from both phases were planned to be pooled to increase sample size.

### Inclusion and exclusion criteria

In- and exclusion criteria were defined to include as many users of OTC preparations for the treatment of LUTS/BPH as possible so that a representative view of the patient population was obtained. Men aged ≥ 50 years were included if they had purchased a non-prescription preparation for the treatment of their own LUTS in a public pharmacy; no medication was administered for study purposes. First time and repeat users were considered. First time users were defined as men who had purchased a medication for the treatment of urination problems for the first time in the past 3 months. Repeat users were defined as all other men, including those purchasing the same product and those switching to another preparation. Consent to participate in the anonymous questionnaire was documented by a dedicated question related to consent for participation and for use of the anonymous data. The only exclusion criterion was for men who purchased a non-prescription medication for the treatment of LUTS for use by a third party, e.g., spouse.

### Ethical committee consultation

The participation in the survey was voluntary and anonymous. There was no treatment as part of the survey, i.e., it was a non-interventional (observational) study. Based on the applicable laws and regulations in Germany, the applicable ethical committee (Ethical Committee of the chamber of physicians of Rhineland-Palatinate) informed us that an ethical vote was neither required nor proposed for this anonymous survey.

### Assessed variables

The questionnaire included the age of the participant, the duration of LUTS, the International Prostate Symptom Score (IPSS) questionnaire, a question about the two most bothersome symptoms captured in the IPSS, and the name of the purchased product. Moreover, multiple choice questions were presented related to expectations regarding the efficacy and tolerability of the product, and reason for purchasing a non-prescription medicine. They were also asked what had alerted them to the specific product they had purchased. Repeat users were additionally asked when the last previous product was purchased, whether the present purchase was the same or a different product, reasons for choosing this product and, finally, whether the participant would recommend the product to a friend with similar complaints.

### Deviations from the pre-specified study protocol

The pre-specified study protocol and the amendments necessary because of the COVID19 pandemic are described in detail in the Online Supplement. Briefly, recruitment of participants from stationary public pharmacies was not feasible during the pandemic. Therefore, recruitment was halted in the fall of 2020. In spring of 2022, a market research organization (Clickworker, Essen, Germany) was involved to start an online survey among its participant pool for the pilot phase, which yielded 84 questionnaires. As this did not indicate a need for modification of the questionnaire, another market research organization (Marktforschung Hopp, Berlin, Germany) was involved that recruited a total of 410 online participants from their pool for the main phase in September 2022. Two additional screening questions were added to the questionnaire to check whether the participant met the inclusion criteria. More detailed information is available in the Online Supplement.

The name of the purchased OTC medication in the free text question indicated that 17 men had obtained a prescription medicine (6 tamsulosin, 3 finasteride, and one each duloxetin, levofloxacin, mirabegron, propiverine, tolterodine or torasemide; two additional men reported use of a dutasteride/tamsulosin fixed-dose combination, and another two use of a medicinal product (pads)). As this was a violation of the inclusion criteria, these 19 participants were excluded from the analyses.

In a small number of cases, not all individual questions of the IPSS questionnaire were completed, which made it impossible to calculate the total IPSS for these men. Moreover, Clickworker reported only accumulated data for individual IPSS questions; due to the complete anonymization, this did not allow the calculation of the individual total IPSS for men in the pilot phase. These two groups were excluded from the analysis of the total IPSS but not for other variables, leaving 384 men with total IPSS scores as opposed to 476–477 men with data on individual IPSS item data.

### Data analysis

Data was analyzed based on a pre-specified statistical analysis plan that was finalized before the first filled questionnaire was received. The participating market research organizations reported anonymized patient data to the investigators as Excel sheets. The datasets from the pilot and main phase were merged by an external pharmacist (Ms. Öykü D. Bese, Ankara, Türkiye).

Based on the exploratory character of the study and in line with recommendations of leading statisticians [6, 7], no hypothesis-testing statistical analysis was performed. Descriptive analyses were performed using Prism 10.1 (GraphPad Software, Los Angeles, CA, USA). Data for categorical variables are presented as absolute numbers and as a percentage of the applicable group; some questions were only directed to repeat users, and answers are presented as percent of that group. Ordinal and continuous variables are shown as median with inter-quartile range (IQR) and/or as means ± SD of n subjects.

## Results

### Patient flow

A total of 86 questionnaires were received in the pilot phase (two on paper sheets from pharmacies, 84 from Clickworker) and 410 in the main phase, i.e., a total of 496 questionnaires. After exclusion of the 19 men, who had obtained a prescription medicine, a total of 477 questionnaires were analyzed. Thus, the minimum number of participants as defined in the study protocol (50 for pilot and 300 for main phase) was exceeded in both phases. The 86 questionnaires from the pilot phase indicated no need for a modification of the questionnaire. Therefore, based on the study protocol, the responses from the pilot phase were included in the overall analysis.

### Demographic data

The participants had a mean age of 64.3 ± 7.8 years (median [IQR] 63 [58; 69]). They reported suffering from LUTS for 3.0 ± 1.9 years (3 [2; 4]). Individual questions of the IPSS questionnaire and the QoL question could be analyzed in 476–477 men. For technical reasons (see above), total IPSS could only be calculated for 384 participants. The mean and median total IPPS was 17.7 ± 7.9 and 17 [11; 24] points, respectively. Results of the individual IPSS questions are shown in Table 1. The QoL question yielded a mean and median score of 3.0 ± 1.3 and 3 [2; 4], respectively. Nocturia (*n* = 216, 45.0%) and a need to empty the bladder again within 2 h (*n* = 96, 20.0%) were reported most frequently as the most bothersome symptom followed by urgency (*n* = 53, 11.7%). All other symptoms were listed by < 10% of the participants as most bothering. A similar picture emerged for the question of the second most bothering symptom, which were frequency (*n* = 133, 27.9%), nocturia (111, 23.3%), incomplete emptying (*n* = 85, 17.9%), urgency (*n* = 67, 14.1%), and weak urine stream (*n* = 48, 10.1%); all other symptoms were listed by < 10% of the participants as second most bothering.

**Table 1:** Results of the IPSS questionnaire shown as medians with IQR and as means ± sd. Note that individual question scores represent 476–477 men, whereas the total IPSS is based on 384 men only (see patients and Methods)

Item	Median [IQR]	Means ± SD
Total symptom score	17 [11; 24]	17.7 ± 7.9
Q 1 (residual urine sensation)	3 [1; 4]	2.6 ± 1.5
Q 2 (frequency)	2 [1; 4]	2.6 ± 1.5
Q 3 (interrupted micturition)	3 [1; 4]	1.9 ± 1.5
Q 4 (urgency)	3 [1; 4]	2.5 ± 1.6
Q 5 (weak stream)	3 [2; 4]	2.9 ± 1.6
Q 6 (straining)	3 [1; 3]	1.9 ± 1.6
Q 7 (nocturia)	3 [2; 4]	3.0 ± 1.6
QoL question	3 [2; 4]	3.0 ± 1.3

### Products used

Participants reported the use of a wide range of preparations. In total, specific LUTS/BPH preparations were identified in 244 men. The most frequently used type was pumpkin seed extracts (*n* = 134), followed by saw palmetto berry (*n* = 10), stinging nettle root extracts (*n* = 5) and pollen (*n* = 1). Combination preparations consisting of extracts of pumpkin seeds and saw palmetto berries (*n* = 73) or pumpkin seeds, saw palmetto and stinging nettle root (*n* = 1) were also used. Complex preparations including more than three and up to 12 ingredients were used by 17 men and homeopathic preparations by two men.

With hindsight, the wording of the inclusion criteria questions in the online survey (originally planned to be applied by the pharmacy staff) may have been insufficiently precise because 59 men reported use of preparations that are not indicated for LUTS/BPH but for other conditions causing LUTS. This included non-prescription preparations for the treatment of urinary tract infections (*n* = 18 plus *n* = 38 for various ‘bladder teas’), overactive bladder (*n* = 2), and prostatitis (*n* = 1). A small number of men reported use of non-prescription preparations not indicated for any urological condition including butylscopolamine (*n* = 2) or one each for bisacodyl, gingko, ginseng, or paracetamol. Moreover, 17 men reported products not indicated for diseases of the lower urinary tract. The other participants did not mention a specific product, or their free-text entry could not be linked to a specific product.

### Efficacy and tolerability expectations

**Fig. 1:**
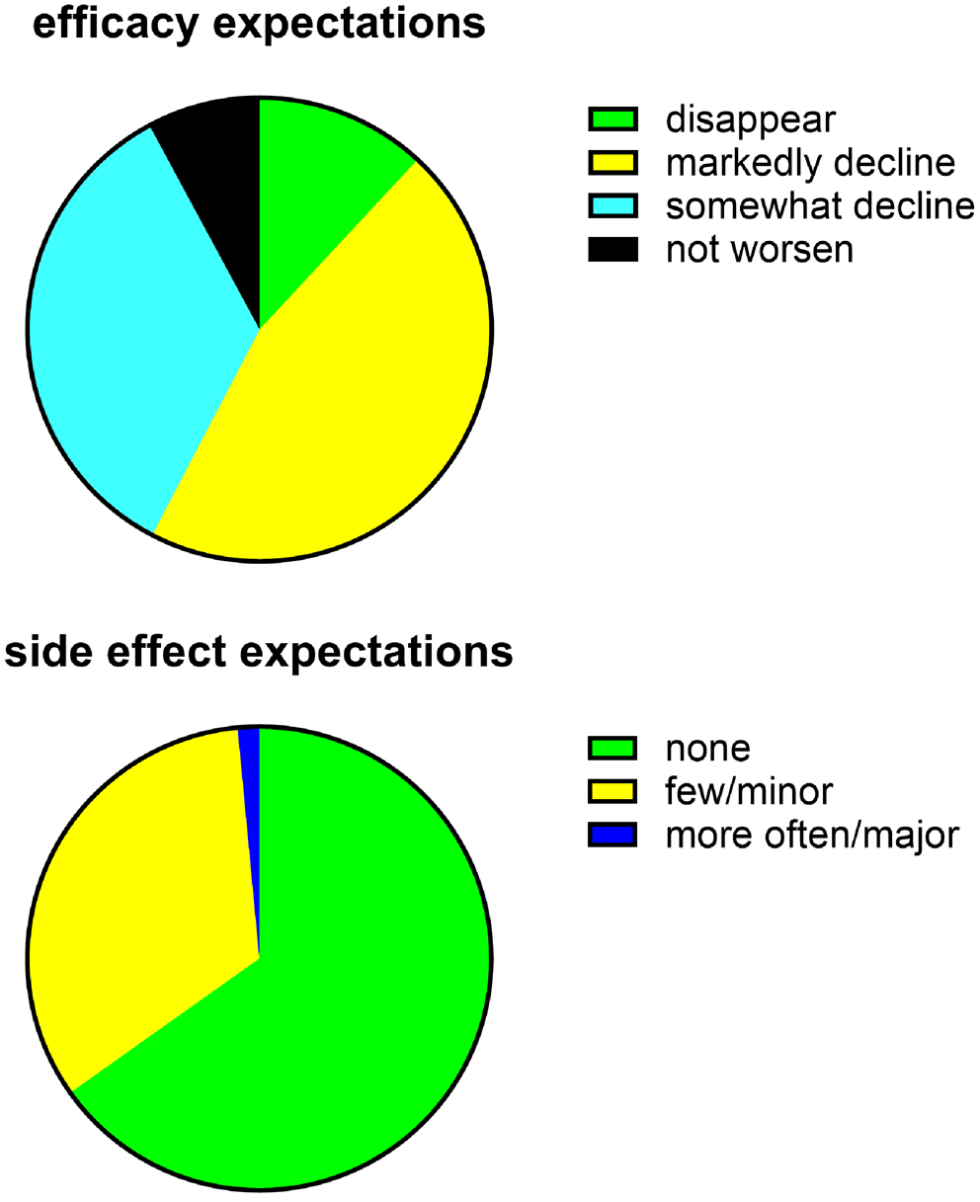
Expectations of efficacy and tolerability based on 470 and 468 responders, respectively

Regarding efficacy, men expected disappearance of their complaints in 11.9%, a relevant decline but not necessarily disappearance in 45.7%, a slight decline in 34.5%, or stable complaints without further increase in symptom severity in 7.9% (Fig. 1). Regarding tolerability, participants expected a lack of side effects in 65.2%, few and minor side effects in 33.3%, and frequent and/or major side effects in 1.5% (Fig. 1).

### Reasons for purchasing OTC product

Seven options were offered to explore the reasons for purchasing a non-prescription treatment in general, from which participants could choose one or more options. A total of 631 responses were received. These included the wish to use a ‘natural’ product (154; 24.4%), the wish to avoid a ‘chemical’ product (99, 15.6%), prior good experience with such products (98, 15.5%), complaints deemed not serious enough to consult a physician (87, 13.8%), recommendation by their doctor (81, 12.8%), being ashamed to talk about complaints (65, 10.3%), and lack of time to visit a doctor (47, 7.4%). Because the wish to use a natural product and to avoid a chemical product are conceptually similar, we calculated a post-hoc non-parametric Spearman rank coefficient, which was 0.3129 [0.2276; 0.3935], indicating a moderate strength of association between these two reasons.

We further asked why a specific product was purchased and offered four options. Eighty-three men (22.1%) reported a recommendation by their physician, 94 (25.1%) a recommendation by the pharmacy personnel, 58 (15.5%) a recommendation by friends, colleagues or family members, and 140 (37.3%) by advertisements.

### Data on repeat users

In total, 171 (36.6%) men were first-time users and 296 (63.3%) repeat users. Among the repeat users, the last purchase of product was reported to have occurred about 3 months ago by 64 (22.9%), about 6 months ago by 72 (25.8%), and about a year or more ago by 144 (51.4%). Most repeat users (297, 94.3%) reported having purchased the same product again. The reasons stated for choosing the specific present product were good experience with it (238, 68.4%), bad experiences with another product (33, 9.5%), and ‘others’ (77, 22.1%). 233 participants (70.4%) stated that they would recommend the product to a friend or family member, whereas 98 (29.6%) did not wish to do so.

## Discussion

This is the first study to characterize men using non-prescription medications for the treatment of their LUTS/BPH. They were similar in age and IPSS to men receiving guideline-recommended prescription medicines in large German cohorts [8–10]. They were most bothered by storage symptoms and especially nocturia. Most study participants expected a marked or reasonable decline of symptoms but not necessarily a disappearance. Reasons to use an OTC product were versatile but the major intention was the wish to use a “natural” and to avoid a “chemical” product. Interestingly, non-prescription drugs in general were recommended by physicians in 12.8% of the study participants, thereby indicating support of OTC-drugs despite recommendations in previous national or international guidelines. Although non-prescription drugs are not reimbursed by health insurance companies in Germany and, therefore, the patient must pay the medication himself, the financial burden for our study participants was not an obstacle. However, our study was not designed to quantify the number of men favoring non-prescription drugs but are unable or unwilling to pay. In addition, our study showed that most men used non-prescription drugs only temporarily > 6 months interval between purchases), indicating the wish to use drugs only for a limited time or only when fluctuating LUTS/BPH appear or are bothersome. The choice of using a specific OTC drug was driven by different reasons among which advertisement for a product was the most important.

### Critique of methods

Lack of feasibility of the original study protocol with patient recruitment via stationary pharmacies during the COVID-19 pandemic required deviations from the study protocol (see Online Supplement). While we used the same questions, recruitment was less targeted: the original plan to recruit via the pharmacy would have assured that only men received the questionnaire who had purchased a product for the self-management of LUTS/BPH. In contrast, the online surveys used proxy questions for LUTS (with specific LUTS/BPH related examples; see Online Supplement) and the purchase of a non-prescription product within the last 3 months. However, this did not exclude participants who had obtained a prescription medicine, a non-prescription medicine or device intended for other urological conditions, or a non-prescription medicine intended for non-urological conditions. Thus, some participants had mistakenly understood the LUTS questions not as related to BPH but rather in a broader sense; this group mainly had used non-prescription medicines for the treatment of urinary tract infections and specifically bladder teas. A specific product for the treatment of LUTS/BPH was identified in 244 men and a product for the treatment of other lower urinary tract conditions in 56 men. Accordingly, approximately 20% of men with an identifiable product obtained preparations for the treatment of LUTS unrelated to BPH.

A small number of men did not answer all IPSS questions, and the data supplied by the agency for the pilot phase provided aggregate data on the individual questions but not on the total IPSS. This may have led to a minor bias for the total IPSS.

### Purchase-related questions

The mean age of participants (64.3 years) and their mean IPSS (17.7 ± 7.9 points) were similar to that of non-interventional studies on prescription medicines in large German cohorts [8–10]. QoL data based on question 8 of the IPSS questionnaire (mean of 3.0 ± 1.3) was also like that from non-interventional studies of prescription medicines. In comparison to data from a survey among patients seeking medical advice for LUTS/BPH, i.e., not necessarily receiving medical treatment, the age and QoL score were also similar but the IPSS score was greater (17 vs. 12) [11], apparently reflecting that men with less severe symptoms less frequently wish to receive treatment. Moreover, the stated symptoms with greatest bother at baseline were similar to those in a large cohort from an academic medical center [12]. Thus, neither a different age nor presence of only mild symptoms can explain why men opted to purchase an OTC product they had to pay for themselves rather than a prescription medicine that their health insurance would pay with little or no co-payment.

Interestingly, randomized placebo-controlled studies for one hexanic saw palmetto berry extract not available in Germany (Permixon^®^) indicated a similar efficacy and tolerability as compared to finasteride [13] or tamsulosin [14]. A large non-interventional study reported that the efficacy and tolerability of this specific saw palmetto extract was similar to that of 5α-reductase inhibitors or α-blockers [15]. A randomized, controlled trial comparing a combination product of saw palmetto and stinging nettle extracts (based on a preparation available in Germany and used by only one man in our survey) also found a comparable efficacy and tolerability compared to tamsulosin [16]. Therefore, it is not surprising that men in our study appeared to have realistic expectations regarding the efficacy and tolerability of the products they obtained, i.e., a marked reduction in LUTS/BPH without or with only minor side effects. Thus, our results were in line with the observations of the non-interventional studies for alfuzosin or tamsulosin [8–10].

Participants provided various reasons why they opted for a non-prescription product they need to pay for themselves. The most frequently mentioned reasons were the wish to use a “natural” product and to avoid a “chemical” product. “Prior positive experience” with an OTC product and “recommendation by a physician” were also relevant reasons in approximately one in six men each. “Being too ashamed of the symptoms” to talk to a doctor was reported by roughly one in seven men. In contrast, “minor severity of symptoms” (in line with the reported IPSS) and “lack of time to visit a doctor” were rarely mentioned. Reasons for selecting a specific product were also heterogeneous; approximately half of patients bought the product because of a recommendation by their physician or pharmacy personnel, and almost 40% due to advertisements; recommendations by friends, colleagues or family members were less important.

About two thirds of participants were repeat users. While a quarter each of participants bought the product 3 and 6 months earlier, approximately half of the participants had done so 12 months or more ago (typical package sizes are for a 3-months treatment). This indicates that only a minor part of men continuously uses the OTC product for the self-management of their LUTS/BPH. This is in line with the fluctuating nature of LUTS/BPH [17–21]. Approximately two thirds of all repeat users chose the same product again due to good experience with it. This may also explain why more than 70% of participants would recommend their product to friends, colleagues or family members.

## Conclusions

The choice of non-prescription medicines for the self-management of LUTS/BPH is not driven by mild symptoms. Rather, general convictions regarding avoidance of chemical and preference for natural products appear to play the key role. These convictions appear so strong that men prefer these OTC treatments despite having to pay for them by themselves and not use prescription treatments that their health insurance would cover. Users have realistic expectations on the efficacy and tolerability of OTC drugs. Most participants were repeat users but only purchased the medicine again in case of reoccurrence of LUTS/BPH. In this case, men most frequently were loyal and chose the same product again.

Declarations.

## Electronic supplementary material

Below is the link to the electronic supplementary material.

Supplementary Material 1

## Data Availability

Raw data are available to qualified investigators upon reasonable request.

## Abbreviations

IPSS: International Prostate Symptom Score
IQR: Interquartile range
LUTS/BPH: Lower urinary tract symptoms suggestive of benign prostatic hyperplasia
OTC: Over-the-counter
QoL: Quality of life
