# Supplementary material for: Characteristics of men using non-prescription medicines to treat their lower urinary tract symptoms
Source: World J Urol. 2025 May 8;43(1):283. doi: 10.1007/s00345-025-05560-1 (PMC12062051; doi:10.1007/s00345-025-05560-1)
Supplement: Supplementary file 1 — Supplementary Material 1 [file 345_2025_5560_MOESM1_ESM.docx]

Online supplement to

**Who are the men taking using non-prescription medicines to treat their LUTS?**

Martin C. Michel, Kurt Miller, Tim Schneider, Christian Ude, Matthias Oelke

The original study protocol as finalized on 20.8.2020 planned the following conduct; it was modified when it became clear that full adherence was impossible during the COVID-19 pandemic. We here present an author translation of the protocol, the original Germany version is provided thereafter.

**Study protocol**

Original version

It was planned to recruit participating public pharmacies by the sponsor (Stiftung Männergesundheit). Each pharmacy was to received an applicable number of printed questionnaires and an information leaflet for the pharmacy staff. Each questionnaire was numbered with noting which pharmacy had received which questionnaire. Linkage of an individual questionnaire to an identifiable patient should be impossible.

The pharmacy staff should contact men who purchased a non-prescription product for self-medication for complaints attributable to LUTS/BPH for anonymous participation in the survey. If interested, the man should receive the questionnaire to be filled at home and be returned postage-free to the sponsor.

The pilot phase should include at least 50 filled questionnaires. They were to be analysed for completeness and possible free text comments. If questionnaires considerably incomplete (more than two unanswered questions) were frequent (more than 10% of all filled questionnaires), the questionnaire should be improved for better understandability, which could involve a reduction in the number of questions and a rewording. An adapted questionnaire was not planned to be reevaluated.

The main phase should include at least 300 filled questionnaires. It was planned to pool the answers from both phases for all questions not adapted after the pilot phase.

**Protocol deviations**

Due to the unfolding COVID-19 pandemic the original protocol was adapted as follows:

- The first round of the pilot phase in stationary pharmacies yielded only two filled questionnaires. Therefore, recruitment was put on hold until autumn of 2020. However, feedback from pharmacies at that time showed that a meaningful conduct of the study as planned was not feasible.
- A second round of recruitment of participants via infomercials in regional media was unsuccessful.
- An alternative pilot phase was conducted online in the period of 3.5.-29.7.2022 by an agency (Clickworker, Essen, Germany). This yielded 84 filled questionnaires that were used as the pilot phase. This did not yield a necessity to adapt the questionnaire. However, one of the filled questionnaires came from a Clickworker employee, and it remains unclear whether this person filled as user or for technical reasons. Removal of this questionnaire was impossible as Clickworker only provided aggregate data.
- Another agency (Marktforschung Hopp, Berlin, Germany) conducted the main phase vom 27.-29.9.2022, which yielded 410 filled questionnaires.
- As the online questionnaires did not allow controlling whether participants belonged to the target group, two screening questions were added. These were “Do you have problems with voiding (e.g., frequent urgency during the day or night; feeling of incomplete bladder emptying; repeatedly stopping and restarting micturition; problems to postpone voiding; weak stream; pressing or straining to void)?” and “Have you purchased a non-prescription (available without a prescription) medicine for your voiding problems in the past 3 months (e.g., in a pharmacy or drug store)?”. Participants not answering both questions with a “yes” received no additional questions and they were not included in the analysis.

A free-text question in the original printed and online questionnaires captured the specific product being purchased. This found that in 17 cases among the online participants in the main phase a prescription medicine was used (6 times tamsulosin, 3 times finasteride, and once each duloxetine, levofloxacin, mirabegron, propiverine, tolterodine, or torasemide; moreover, two men used a dutasteride/tamsulosin combination product) and two mean a medicinal product (pads). The questionnaires of these 19 participants were excluded from the analysis because of violation of the inclusion criteria.

In few cases not all individual IPSS questions were answered by the participants, which did not allow calculation of a total IPSS score. On the other hand, Clickworker only provided accumulated data for individual IPSS questions, which also did not allow calculation of a total IPSS score.

The filled questionnaires were planned to be digitized by the sponsor and sent to the principal investigator. The principle investigators (if needed with support from qualified personnel) should convert the questionnaires into an electronic format and perform an aggregate analysis. In deviation from the original protocol, the results from the online survey were directly entered into Excel files by the agencies.

The staff of the stationary pharmacies could directly determine, who had purchase a non-prescription product for own use. The online surveys included two additional questions to reach the same purpose (screen out questions).

During analysis of the questionnaires, it emerged that some participants had obtained no non-prescription but a prescription medicine. Their responses were excluded from the analysis.

The merger of the three rounds (paper version in pharmacies, Clickworker, Hopp) was conducted by a pharmacist (Ms. Öykü Bese, Ankara, Türkiye).

Based on the exploratory character of the study, no hypothesis-testing statistical analysis was conducted and online descriptive analysis was performed. To this end, data from the merged Excel files were transferred to Prism 10.1 (GraphPad Software, Los Angeles, CA, USA). The data for categorical variables were presented as absolute numbers and as % of the applicable group; some questions targeted repeat users and are expressed as % of repeat users. The ordinal and continues variables were presented as median with interquartile ranges and/or as means ± standard deviation of n participants.

**Original German version**

Teilnehmende öffentliche Apotheken sollten durch den Sponsor rekrutiert werden und eine angemessene Anzahl von Fragebögen sowie ein Informationsblatt für ihre Mitarbeiterinnen und Mitarbeiter erhalten. Die Fragebögen sollten nummeriert, und es sollte vermerkt werden, welche Fragebögen an welche Apotheken geliefert wurden. Eine Zuordnung einzelner Fragebogen zu identifizierbaren Patienten sollte unmöglich sein.

Das Apothekenpersonal sollte Männer ansprechen, die ein rezeptfreies Präparat zur Selbstmedikation von Beschwerden im Sinne eines BPS für den eigenen Gebrauch gekauft haben und sie einladen, an der anonymen Befragung teilzunehmen. Bei Interesse sollte der Mann einen Fragebogen erhalten, den er zuhause ausfüllen und portofrei an den Sponsor zurückschicken sollte.

Die Pilotphase sollte mindestens 50 ausgefüllte Fragebögen enthalten. Diese sollten ausgewertet werden unter dem Gesichtspunkt der Vollständigkeit und der freien Kommentare. Falls sehr unvollständig ausgefüllte Fragebögen (mehr als 2 Fragen unbeantwortet) häufig sein sollten (mehr als 10% der ausgefüllten Bögen), sollte der Fragebogen mit dem Ziel einer besseren Verständlichkeit überarbeitet werden, was sowohl eine Verminderung der Fragen als auch eine Umformulierung der Fragen beinhalten konnte. Ein eventuell überarbeiteter Fragebogen sollte nicht erneut erprobt werben.

Die Hauptphase sollte mindestens 300 ausgefüllte Fragebögen enthalten. Für alle in beiden Phasen verwendeten und nicht geänderten Fragen sollten die Antworten aus beiden Phasen in die Auswertung aufgenommen werden.

## Deviations from original study protocol

Wegen der sich entfaltenden COVID19 Pandemie musste vom Protokoll vom 20.8.2020 in folgender Weise abgewichen werden:

- Die erste Runde in stationären Apotheken hatte einen Rücklauf von nur 2 Fragebögen. Deshalb wurde die Rekrutierung bis zum Herbst 2020 angehalten, aber auch dann ergab Feedback aus Apotheken, dass eine sinnvolle Durchführung nicht möglich war.
- Eine zweite Runde mit Rekrutierung von Teilnehmern über Infomercials in regionalen Medien blieb ebenfalls erfolglos.
- Es wurde dann für die Pilotphase über eine Agentur (Clickworker, Essen) in der Zeit vom 3.5.2022 bis 29.7.2022 eine online Umfrage gestartet. Diese ergab 84 Rückläufe, die für die Pilotphase verwendet wurden. Daraus ergab sich keine Notwendigkeit, den Fragebogen anzupassen. Einer dieser Rückläufe war von einem Clickworker-Mitarbeiter, und es bleibt unklar, ob er in seiner Funktion als Verwender oder aus technischen Gründen Daten eingegeben hat. Eine Entfernung dieser Antworten war nicht möglich, da Clickworker nur aggregierte Daten zur Verfügung gestellt hat.
- Über eine andere Agentur (Marktforschung Hopp, Berlin) wurde in der Zeit vom 27.-29.9.2022 eine online Umfrage für die Hauptphase durchgeführt, die insgesamt 410 Rückläufe ergab.
- Da bei den online-Fragen nicht möglich war, zu kontrollieren, ob die Teilnehmer zur Zielgruppe gehörten, wurden zwei Screening-Fragen eingeführt. Diese lauteten „Haben Sie Beschwerden beim Wasserlassen (z.B. häufiger Harndrang am Tag oder in der Nacht; Gefühl, dass Ihre Blase nach dem Wasserlassen nicht ganz entleert ist; beim Wasserlassen mehrmals aufhören und neu beginnen müssen; Schwierigkeiten, das Wasserlassen hinauszuzögern; schwacher Strahl beim Wasserlassen; pressen oder sich anstrengen müssen, um mit dem Wasserlassen zu beginnen)?“ und „Haben Sie in den vergangenen 3 Monaten ein rezeptfreies (frei verkäufliches) Mittel gegen Ihre Beschwerden beim Wasserlassen erworben (z.B. in der Apotheke oder in einer Drogerie)?“ Teilnehmer, die nicht beide Fragen mit „ja“ beantworteten, wurden keine weiteren Fragen gestellt und sie wurden nicht in die Auswertung übernommen.

Eine offene Frage im ursprünglichen schriftlichen und in den online-Fragebögen erfasste das erworbene Präparat. Dabei ergab sich in 17 Fällen der online rekrutierten Teilnehmer, dass das verwendete Präparat einen verschreibungspflichtigen Wirkstoff enthielt (6 mal Tamsulosin, 3 mal Finasterid, sowie je 1 mal Duloxetin, Levofloxacin, Mirabegron, Propiverin, Tolterodin oder Torasemid; darüber hinaus wurde in zwei Fällen ein Kombinationspräparat aus Dutasterid plus Tamsulosin angegeben) und in zwei Fällen, dass es sich um ein Medizinprodukt handelte (Vorlagen). Die Antworten dieser 19 Teilnehmer wurden aus der Analyse ausgeschlossen, da sie nicht den Einschlusskriterien entsprachen.

In wenigen Fällen wurden nicht alle Einzelfragen zum IPSS von den Teilnehmern beantwortet; in diesen Fällen konnte kein IPSS-Gesamtscore berechnet werden. Von Clickworker wurden nur akkumulierte Daten zu den Einzelfragen des IPSS zur Verfügung gestellt, was ebenfalls keine Berechnung des Gesamtscores zuließ.

Die ausgefüllten Fragebögen sollten vom Sponsor digitalisiert und dem Studienleiter zugestellt werden. Der Studienleiter sollte (ggf. mit Unterstützung von einschlägig qualifizierten Mitarbeitern) die Antworten der Fragebögen in ein elektronisches Format überführen und zusammenfassen. Der Studienleiter und seine Mitarbeiter sollten diese auf Basis des SAP analysieren. In Abweichung vom Studienprotokoll wurden die Ergebnisse der online Befragungen direkt von der jeweiligen Agentur in Excel-Dateien dokumentiert.

In den Präsenzapotheken konnte das Apothekenpersonal direkt feststellen, wer ein nicht verschreibungspflichtiges Präparat für den eigenen Bedarf erworben hatte. In den online Befragungen wurden zwei zusätzliche Fragen implementiert, die dies sicherstellten (screen out Fragen).

Bei der Auswertung der Fragen stellte sich heraus, dass in den online Befragungen einige Teilnehmer kein freiverkäufliches, sondern ein verschreibungspflichtiges Präparat erworben hatten. Die Antworten dieser Patienten wurden aus der Analyse ausgeschlossen.

Die Zusammenführung der Daten aus den drei Datensätzen (Papier, Clickworker, Hopp) erfolgte durch eine approbierte Apothekerin (Frau Öykü Bese, Ankara, Türkei).

Basierend auf dem explorativen Charakter der Studie wurden keine Hypothesen-testenden Statistikverfahren angewandt. Vielmehr wurden nur deskriptive Analysen durchgeführt. Dies geschah nach Überführungen der integrierten Daten aus den Excel-Dateien mit Prism 10.1 (GraphPad Software, Los Angeles, CA, USA). Die Daten für kategorische Variablen werden präsentiert als absolute Zahlen und als % der zugrundeliegenden Gesamtgruppe; einige Fragen richteten sich nur an Wiederverwender und werden als % der Wiederverwender präsentiert. Die ordinalen und kontinuierlichen Variablen werden als Median mit Interquartilen (IQR) und/oder als Mittelwert ± Standardabweichung von n Teilnehmern präsentiert.
